# Household Risk Factors for Colonization with Multidrug-Resistant *Staphylococcus aureus* Isolates

**DOI:** 10.1371/journal.pone.0054733

**Published:** 2013-01-24

**Authors:** Meghan F. Davis, Amy E. Peterson, Kathleen G. Julian, Wallace H. Greene, Lance B. Price, Kenrad Nelson, Cynthia J. Whitener, Ellen K. Silbergeld

**Affiliations:** 1 Department of Environmental Health Sciences, Johns Hopkins Bloomberg School of Public Health, Baltimore, Maryland, United States of America; 2 Department of Epidemiology, Johns Hopkins Bloomberg School of Public Health, Baltimore, Maryland, United States of America; 3 Penn State Hershey Medical Center, Hershey, Pennsylvania, United States of America; 4 Division of Pathogen Genomics, The Translational Genomics Research Institute (TGen North), Phoenix, Arizona, United States of America; Rockefeller University, United States of America

## Abstract

Antimicrobial resistance, particularly in pathogens such as methicillin-resistant *Staphylococcus aureus* (MRSA), limits treatment options and increases healthcare costs. To understand patient risk factors, including household and animal contact, potentially associated with colonization with multidrug-resistant MRSA isolates, we performed a prospective study of case patients colonized with MRSA on admission to a rural tertiary care hospital. Patients were interviewed and antimicrobial resistance patterns were tested among isolates from admitted patients colonized with MRSA in 2009–10. Prevalence of resistance was compared by case-patient risk factors and length-of-stay outcome among 88 MRSA case patients. Results were compared to NHANES 2003–04. Overall prevalence of multidrug resistance (non-susceptibility to ≥four antimicrobial classes) in MRSA nasal isolates was high (73%) and was associated with a 1.5-day increase in subsequent length of stay (*p* = 0.008). History of hospitalization within the past six months, but not antimicrobial use in the same time period, was associated with resistance patterns. Within a subset of working-age case patients without recent history of hospitalization, animal contact was potentially associated with multidrug resistance. History of hospitalization, older age, and small household size were associated with multidrug resistance in NHANES data. In conclusion, recent hospitalization of case patients was predictive of antimicrobial resistance in MRSA isolates, but novel risk factors associated with the household may be emerging in CA-MRSA case patients. Understanding drivers of antimicrobial resistance in MRSA isolates is important to hospital infection control efforts, relevant to patient outcomes and to indicators of the economic burden of antimicrobial resistance.

## Introduction

Methicillin-resistant *Staphylococcus aureus* (MRSA) is a pandemic antimicrobial-resistant pathogen [1]. In 2004, an estimated 1.5% of the United States population, or approximately 4 million people, were nasally colonized with MRSA [2]. Nasal colonization increases risk for development of clinical infection [3]. Antimicrobial-resistant pathogens, which include MRSA, have human costs in morbidity and mortality, and they have been estimated to have healthcare costs in excess of $4 billion annually in the U.S. [4]. As a result, understanding the epidemiology of multidrug-resistant MRSA case-patients is both clinically and economically relevant to healthcare surveillance and control efforts.

MRSA epidemiology in the United States is shifting rapidly, as strains historically considered community-associated enter hospitals, and hospital strains disseminate into the community [5]–[8]. However, some authors have suggested that isolate antimicrobial susceptibility may continue to distinguish community-acquired (CA-)MRSA isolates from those acquired in the hospital, and that isolate resistance to certain antimicrobials (ciprofloxacin, clindamycin, and aminoglycosides) may typify hospital-acquired (HA-)MRSA isolates [9]–[12]. In addition, new risk factors for acquisition of MRSA may be emerging. Human households [13]–[15] and animals [16], [17] recently have been described as potential community reservoirs for MRSA.

To describe case-patient epidemiology and evaluate novel household and animal risk factors as potential drivers of antimicrobial resistance, we interviewed MRSA positive case patients identified from nasal colonization surveillance at a tertiary care center serving largely rural and suburban communities. MRSA isolates were tested for antimicrobial susceptibility. We also evaluated associations between isolate drug resistance and subsequent length-of-stay (LOS) among case-patients, using LOS as an economic and human cost marker for potential associations between drug resistance and factors related to hospitalization. Risk factor results were compared to data from MRSA-colonized participants in the National Health and Nutrition Examination Survey (NHANES) 2003–04.

## Methods

### Research Design

We enrolled patients over the age of 18 years at Penn State Hershey Medical Center (PSHMC) between August 2009 and March 2010 as previously described [18]. As part of a larger case-control study, MRSA case patients identified on admission via screening nasal swabs were interviewed as a prospective case cohort to characterize MRSA isolates by multi-locus sequence typing (MLST) and antimicrobial susceptibility patterns from a hospital source population that included predominantly rural and suburban communities [18]. This manuscript is limited to analysis of the case-patients from whom a MRSA isolate was available for antimicrobial susceptibility testing and who had complete data for all risk factors.

### Survey

Patients were interviewed for self-reported risk factors that included demographic information; hospitalization within the past month, six months, or year prior to admission for themselves and for family members; antimicrobial drug use within the past month, six months or year prior to admission for themselves and for family members; animal contact, including livestock (cows, pigs, and poultry); household pet ownership (dogs and cats only); and number of people living in the household. Subsequent length of stay was determined through record review.

### Sample Collection

Swabs of the anterior nares of patients were collected within 48 hours of admission and these swabs were processed at the PSHMC virology laboratory using the BD GeneOhm™ MRSA Assay (Becton Dickinson Diagnostics, San Diego, CA). MRSA-positive swabs by this PCR method were archived and subsequently cultured for viable MRSA isolates using commercial MRSA Select™ agar plates (Bio-Rad Laboratories, Hercules, CA). Isolates were confirmed as MRSA using a real-time PCR assay by detection of *mecA* and *femA* genes (Pathogene, LLC). Due to potential presence of variant *mecA* genes, MSSA isolates found to be beta-lactam resistant were tested for presence of *mecC* using a newly designed universal primer as previously described [19], [20].

### Antimicrobial Susceptibility Testing

MRSA and MSSA isolates were tested for antimicrobial susceptibility using disc diffusion methods [21], [22], including erythromycin-induced resistance to clindamycin (D-test), following CLSI guidelines [23] to nine antimicrobials: chloramphenicol, quinupristin/dalfopristin (Synercid), tetracycline, gentamicin, amikacin, trimethoprim/sulfamethoxazole, clindamycin, ciprofloxacin, and erythromycin. Multi-drug resistance (MDR4) was defined as beta-lactam resistance by *mecA* gene presence plus nonsusceptibility (inducible, intermediate or high-level resistance) to three additional classes of antimicrobial drugs by disc diffusion methods, based on a definition reported by SENTRY [24]. An additional category of high multidrug resistance (MDR5) was included to evaluate whether risk factors differed for isolates more difficult to treat, and this was defined as beta-lactam resistance (*mecA* gene presence, all isolates by definition) plus high-level (complete) resistance to four additional classes of antimicrobial drugs (*i.e.,* resistance to ≥ five antimicrobials). For MDR5 and for individual antimicrobial drug evaluation, risk factors were compared to high-level (complete) resistance only, including intermediate with susceptible isolates in models, because high-level resistance may be associated with a higher probability of acquired resistance [25], as opposed to resistance based on other mechanisms, *e.g.* via multiple mutations in cell wall biosynthesis. For clindamycin, inducible resistance was included with high-level resistance phenotypes [26].

All isolates were screened for vancomycin resistance by real-time PCR assay for the *vanA* gene (Pathogene, LLC) and disc diffusion testing. Because of previous findings of hVISA isolates in this patient population [27], 33 of the isolates were selected for further vancomycin susceptibility testing on the basis of their susceptibility profiles (*e.g.* quinupristin/dalfopristin non-susceptibility or MDR4). These isolates were screened using a standard VA E-test and also a GRD E-test (vancomycin and teicoplanin) for potential hGISA phenotype as previously described [27]. Isolates with positive GRD E-tests [28] were tested using a population analysis as previously described [27]. Briefly, 10^7^ and 10^6^ inoculations were placed on agar plates containing 1, 2, 4, 7, and 8 ug/ml vancomycin. Growth at the level of 4 ug/ml with a 10^6^ inoculation was considered indicative of hGISA positivity. Results were validated against quality control strains ATCC 29213, ATCC Mu3 (hGISA) and ATCC Mu50 (GISA).

### Statistical Analysis

We estimated unadjusted and adjusted associations for antimicrobial resistance by risk factor using prevalence ratios (PRs). We calculated PRs using Poisson models with robust estimation of standard errors as described previously [29], [30] using Stata 11 (College Station, TX). P-values ≤0.05 were considered statistically significant, and p-values ≤0.10 were considered to approach statistical significance.

*A priori*, covariates included self-reported age, gender, race, history of hospitalization, prior use of antimicrobials, exposure to animals, and household size. Categorical dummy variables for hospitalization or antimicrobial use within one month compared to within six months were created, assigning 0 if patients self-reported no hospital contact or antimicrobial use within six months, assigning 1 if patients self-reported hospitalization or antimicrobial use within six months prior to admission, and 2 if patients self-reported hospitalization or antimicrobial use within a month of admission. The six-month cut-off and definitions for HA- versus CA-MRSA assignment were selected based on prior work in this study population [18]. Self-reported contact with dogs and cats was colinear with self-reported household pet ownership; hence these variables were aggregated. Due to small numbers, livestock contact was aggregated from individual reporting of pig, poultry, or cow contact. Age, household size, and animal exposure variables were dichotomized. Because of the small numbers with non-white race (*n = 6*), this risk factor was not examined further.

Linear regression models were run with log-transformed length of stay (LOS) as an outcome, evaluating potential association with antimicrobial resistance patterns. Beta coefficients from log-transformed models were exponentiated to return a point estimate, in days, for average LOS increase in patients colonized with MDR isolates.

The Penn State Hershey Medical Center and Johns Hopkins Bloomberg School of Public Health Institutional Review Boards reviewed and approved this study. Patients provided written informed consent to participate in the study.

### NHANES Analysis

To provide a descriptive comparison between this geographically-limited study of hospital inpatients and data from a wider U.S. population, statistical analysis was run on a subset of all MRSA-colonized participants in NHANES 2003–04, which represented the most recent national data available to the public on MRSA colonization [31]. Risk factor data available in NHANES 2003–04 included gender, age, self-reported history hospitalization within the past year, and household size [31]. Antimicrobial use data was available only for the prior one month; this variable was not included in analysis due to the inconsistent time frame with the hospitalization variable. Data on animal contact or pet ownership was not available. Methods for antimicrobial susceptibility testing in NHANES previously have been described [32]. Identical analysis was run on the NHANES data and the PSHMC datasets for descriptive comparison. Trends also were evaluated for *S. aureus* nasal colonization and identified risk factors in NHANES. Survey weighting was not used for NHANES models limited to MRSA-positive individuals due to the small sample size.

## Results

### Case-patient Selection

**Figure 1:**
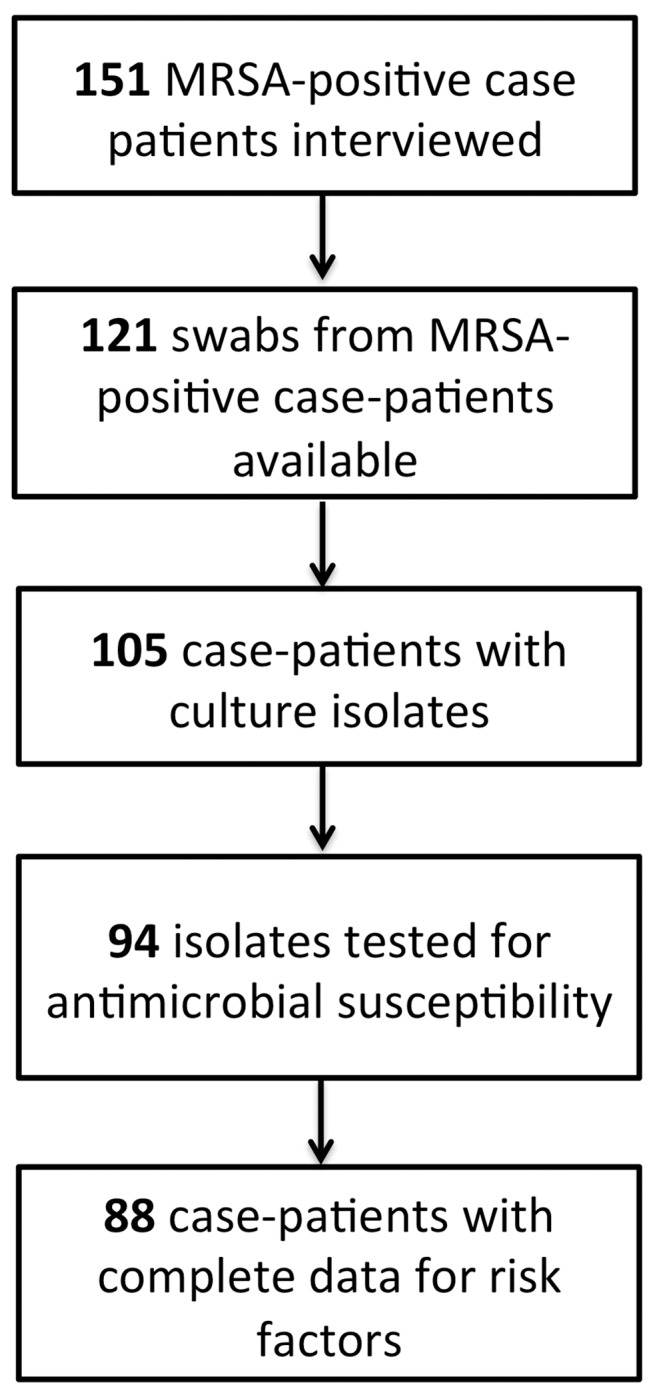
Study design for analysis of risk factors from case-patients interviewed at Penn State Hershey Medical Center.

**Figure 1** presents the selection process for case-patient inclusion in these analyses. Analysis was restricted to 88 individuals for whom data was complete for risk factors and from whom isolates were available for antimicrobial susceptibility testing. Epidemiologic comparison of the 63 case patients not included in this analysis demonstrated that these patients did not differ significantly in demographic characteristics, rates of prior hospitalization, or self-reported antimicrobial use as the included 88 case patients.

### Prevalence of Antimicrobial Resistance

Overall, the prevalence of inducible and high-level resistance to individual antimicrobial drugs among the 88 isolates was: erythromycin, 90%; ciprofloxacin, 82%; clindamycin, 58%; amikacin, 24%; trimethoprim-sulfamethoxazole, 15%; gentamicin, 10%; tetracycine, 7%; quinupristin/dalfopristin (Synercid), 1%; and chloramphenicol, 1%.

All isolates were negative for *vanA* by real-time PCR and were susceptible on vancomycin disc diffusion testing. Based on antimicrobial susceptibility profile screening, we selected a subset of 33 isolates to evaluate further. All were classified as vancomycin-susceptible based on a MIC of 2 or less by standard vancomycin E-test analysis. However, 16 (48%) were considered suspect for heterogeneous glycopeptide intermediate resistant *S. aureus* (hGISA) phenotype by vancomycin-teicoplanin (GRD) E-test. One (6%) of these 16 isolates was confirmed as a heterogeneous glycopeptide intermediate resistant *S. aureus* (hGISA) on the basis of population analysis, and details on this case are described below. Due to the low prevalence in this cohort (*n = 1*), vancomycin, quinupristin-dalfopristin, and chloramphenicol resistance were excluded from further analysis.

### Case Report

This paper reports on the finding of a methicillin-resistant and heterogeneous glycopeptide-intermediate *S. aureus* (hGISA) isolate, typed as a ST5 (CC5) on the basis of multi-locus sequence testing [33], from a nasally colonized 76-year old Caucasian female case-patient who reported no personal or household member (*n* = 1) history of hospitalization, antimicrobial drug use, or healthcare occupational contact in the past year and who did not currently reside in a nursing home or have home nursing care on the basis of record review. She did have routine outpatient contact for follow up of chronic medical conditions. Her isolate was highly resistant to all nine antimicrobials tested and represented the only strain we identified with high-level resistance to quinupristin-dalfopristin and chloramphenicol. Although CC5 isolates historically have been associated with healthcare acquisition, recent reports have demonstrated that these strains may be establishing circulation patterns in the community [7], and the finding of such a highly-drug resistant isolate in a patient with CA-MRSA epidemiology is notable.

### Prevalence of Antimicrobial Resistance by Risk Factor

**Table 1** presents prevalence for individual resistance patterns and for multidrug resistance (MDR4 and MDR5) according to self-reported variables for gender, age, history of hospitalization and antimicrobial use, animal contact (livestock and household pets separately), and human household size for the 88 case-patients. The rate of multidrug resistance (MDR4) was high overall (73%). Highly multidrug-resistant isolates (MDR5) comprised almost a quarter of isolates. Almost half (48%) of case-patients reported a history of hospitalization within the prior six months, and antimicrobial use in the same period (68%) frequently was found.

**Table 1:** Prevalences of antimicrobial resistance by risk factor among 88 MRSA isolates from Penn State Hershey Medical Center admitted patients, August 2009 to February 2010.

	Multidrug Resistance (MDR4)	High Multidrug Resistance (MDR5)	Ciprofloxacin Resistant (CIPR)	Clindamycin Resistant (CLIR)	Amikacin Resistant (AMKR)
	*4+ classes of non-susceptibility*	*5+ classes of* *high-level* *resistance*	*high-level resistance*	*high-level resistance*	*high-level resistance*
**Overall, N (%)**	**64 (73%)**	**20 (23%)**	**72 (82%)**	**51 (58%)**	**21 (24%)**
**Gender**					
Female, *n* = 35	25 (71%)	11 (31%)	29 (83%)	20 (57%)	10 (29%)
Male (*ref*), *n* = 53	39 (74%)	9 (17′%)	43 (81%)	31 (58%)	11 (21%)
**Age**					
65 years or older, *n* = 38	30 (80%)	10 (26%)	35 (92%)	26 (68%)	7 (19%)
Under 65 (*ref*), *n* = 50	34 (68%)	10 (20%)	37 (74%)	25 (50%)	14 (28%)
**Hospitalization**					
Within 1 month, *n* = 24	20 (83%)	9 (38%)	22 (92%)	17 (71%)	8 (33%)
1–6 mo, *n* = 18	17 (94%)	5 (28%)	17 (94%)	13 (72%)	2 (11%)
Over 6 mo (*ref*), *n* = 46	27 (59%)	6 (13%)	33 (72%)	21 (46%)	11 (24%)
**Antimicrobial use**					
Within 1 month, *n* = 36	31 (86%)	11 (31%)	33 (92%)	24 (67%)	10 (28%)
1–6 mo, *n* = 24	18 (75%)	4 (17%)	20 (83%)	13 (54%)	6 (25%)
Over 6 mo (*ref*), *n* = 28	15 (54%)	5 (18%)	19 (68%)	14 (50%)	5 (18%)
**Livestock Exposure**					
Direct contact, *n* = 12‡	7 (58%)	1 (8%)	8 (67%)	7 (58%)	3 (25%)
No direct contact (*ref*), *n* = 76	57 (75%)	19 (25%)	64 (84%)	44 (58%)	18 (24%)
**Household pets**					
Have pets, *n* = 49‡‡	39 (80%)	12 (24%)	40 (82%)	30 (61%)	12 (24%)
Don’t have pets (*ref*), *n* = 39	25 (64%)	8 (21%)	32 (82%)	21 (54%)	9 (23%)
**Household Size** *					
Over 2, *n* = 32	24 (75%)	8 (25%)	23 (72%)	18 (56%)	9 (28%)
2 or fewer (*ref*), *n* = 56	40 (71%)	12 (21%)	49 (88%)	33 (59%)	12 (21%)

N (%) shown are for the resistant population compared to the susceptible population. Intermediates are included with resistant isolates for the SENTRY MDR definition, but are included with the susceptible population for the remainder of the categories. Race was not included due to small numbers of non-white participants (N = 6).

‡ Six case patients reported living on a farm; six reported farm occupation; two reported chicken contact, six reported cow contact; and one reported pig contact (categories non-exclusive).

‡‡ 34 dogs and 22 cats.

* Household size includes index patient.

Three antimicrobials–clindamycin, ciprofloxacin, and amikacin–were selected to demonstrate trends according to risk factor in part based on the potential utility of these resistance patterns to differentiate isolates as HA-MRSA or CA-MRSA [9]–[11]. Erythromycin was excluded from analysis due to extremely high resistance prevalence, and others were excluded due to low prevalence, which led to unstable model estimates (zero strata).

### Unadjusted and Adjusted Models

**Table 2** presents unadjusted and adjusted model estimates of prevalence ratios for each resistance pattern by risk factor. In adjusted models, history of hospitalization was associated with multidrug resistance, clindamycin resistance, and ciprofloxacin resistance, controlling for the effects of other covariates. Such associations with hospitalization were not present for amikacin resistance; instead, history of hospitalization in the one to six months prior to admission had a prevalence ratio (PR) of 0.31, although this association was not significant (*p* = 0.09). A sensitivity analysis including self-reported information on healthcare occupation reported by the case patient or a household member as recent (≤1 mo) hospital contact produced no significant changes in inference, although estimates of association strengthened slightly in all cases.

**Table 2:** Unadjusted and adjusted prevalence ratios for antimicrobial resistance by risk factor among 88 MRSA isolates from Penn State Hershey Medical Center admitted patients, August 2009 to February 2010.

	Multidrug Resistance (MDR4)					High Multidrug Resistance (MDR5)				Ciprofloxacin Resistant (CIPR)				Clindamycin Resistant (CLIR)			Amikacin Resistant (AMKR)		
	*4+ classes of* *non-susceptibility*					*5+ classes of* *high-level resistance*				*high-level resistance*				*high-level resistance*			*high-level resistance*		
	PR		95% CI	p-value		PR		95% CI	p-value	PR		95% CI	p-value	PR	95% CI	p-value	PR	95% CI	p-value
**Gender (male is ref)**																			
Unadjusted	0.97		0.74–1.27	0.83		1.85		0.85–4.02	0.12	1.02		0.84–1.25	0.84	0.98	0.68–1.41	0.90	1.38	0.65–2.90	0.40
Adjusted	0.96		0.73–1.26	0.78		1.70		0.79–3.64	0.18	1.00		0.82–1.21	0.97	0.91	0.63–1.31	0.61	1.58	0.74–3.38	0.24
**Age (under 65 is ref)**																			
Unadjusted	1.16		0.90–1.49	0.25		1.32		0.61–2.85	0.49	**1.24**		**1.03–1.50**	**0.02**	*1.37*	*0.96–1.95*	*0.08*	0.66	0.29–1.48	0.31
Adjusted	1.23		0.93–1.61	0.15		1.12		0.51–2.47	0.78	**1.24**		**1.02–1.51**	**0.03**	*1.42*	*0.98–2.06*	*0.07*	0.58	0.26–1.27	0.17
**Hospitalization (>6** **mo is ref)**																			
Within 1 month, unadjusted	**1.42**		**1.05–1.92**	**0.02**		**2.88**		**1.15–7.16**	**0.02**	**1.28**		**1.03–1.59**	**0.03**	**1.55**	**1.03–2.34**	**0.04**	1.39	0.65–3.01	0.40
Within 1 month, adjusted	1.14		0.79–1.65	0.49		**3.15**		**1.16–8.57**	**0.03**	1.12		0.88–1.44	0.36	1.42	0.82–2.45	0.22	1.28	0.45–3.62	0.64
1–6 months, unadjusted	**1.61**		**1.23–2.10**	**0.001**		2.13		0.74–6.15	0.16	**1.32**		**1.06–1.63**	**0.01**	**1.58**	**1.03–2.43**	**0.04**	0.46	0.11–1.91	0.29
1–6 months, adjusted	**1.46**		**1.09–1.96**	**0.01**		*2.38*		*0.85–6.67*	*0.10*	**1.28**		**1.02–1.62**	**0.04**	**1.72**	**1.06–2.79**	**0.03**	*0.31*	*0.08–1.19*	*0.09*
**Antimicrobial use (>6** **mo is ref)**																			
Within 1 month, unadjusted	**1.61**	**1.11–2.33**			**0.01**	1.71	0.67–4.38		0.26	**1.35**	**1.03–1.78**		**0.03**	1.33	0.86–2.07	0.20	1.56	0.60–4.06	0.37
Within 1 month, adjusted	*1.42*	*0.93–2.15*			*0.10*	0.84	0.29–2.46		0.75	*1.31*	*0.98–1.75*		*0.06*	1.10	0.61–1.97	0.75	1.51	0.46–4.99	0.50
1–6 months, unadjusted	1.40	0.92–2.13			0.11	0.93	0.28–3.11		0.91	1.23	0.90–1.68		0.20	1.08	0.64–1.83	0.77	1.40	0.48–4.04	0.53
1–6 months, adjusted	1.16	0.74–1.82			0.51	0.57	0.18–1.84		0.35	1.15	0.83–1.59		0.42	0.81	0.45–1.45	0.47	1.74	0.57–5.34	0.33
**Livestock Exposure (no contact is ref)**																			
Bivarite	0.78	0.47–1.28			0.32	0.33	0.04–2.29		0.26	0.79	0.52–1.20		0.27	1.01	0.60–1.69	0.98	1.06	0.36–3.07	0.92
Adjusted	0.79	0.53–1.17			0.24	0.31	0.04–2.39		0.26	0.81	0.57–1.15		0.24	1.03	0.63–1.67	0.92	0.93	0.31–2.79	0.89
**Household pets (no pets is ref)**																			
Unadjusted	1.24	0.94–1.64			0.12	1.19	0.54–2.64		0.66	0.99	0.82–1.21		0.96	1.14	0.79–1.64	0.49	1.06	0.50–2.27	0.88
Adjusted	1.23	0.93–1.61			0.14	1.12	0.49–2.55		0.79	1.03	0.84–1.24		0.80	1.07	0.73–1.57	0.71	0.83	0.37–1.88	0.66
**Household Size** * **(2 or fewer is ref)**																			
Unadjusted	1.05	0.81–1.36			0.71	1.17	0.53–2.56		0.70	0.82	0.65–1.04		0.11	0.95	0.65–1.39	0.81	1.31	0.62–2.78	0.48
Adjusted	0.93	0.73–1.20			0.59	1.05	0.49–2.27		0.90	**0.79**	**0.63–0.99**		**0.04**	0.94	0.64–1.38	0.51	1.42	0.68–2.96	0.35

Intermediates are included with resistant isolates for the SENTRY MDR definition, but are included with the susceptible population for the remainder of the categories. Unadjusted and adjusted results are limited to the 88 individuals for whom complete data on all potential covariates is available. Adjusted models control for gender, age, history of hospitalization, history of antimicrobial use, livestock exposure, household pets, and household size. Prevalence ratios (PRs) shown are estimated from poisson regression models (categorical models used for hospitalization and antibiotic use). Significant associations (two-sided p<0.05) are shown in bold. Associations that are non-significant but approach significance (two-sided p<0.10) are italicized. Race was not included due to small numbers of non-white participants (*n* = 6).

* Household size includes index patient.

Estimates of association between resistance patterns and antimicrobial use were weaker, and attenuated to non-significance in adjusted models in all cases. When sensitivity analysis was performed including intermediate with high-level (complete) resistance, antimicrobial use in the previous month was significantly associated with risk for resistance to ciprofloxacin or to multidrug resistance (MDR4), and antimicrobial use remained a significant predictor in adjusted models. In these adjusted models, estimates of association with hospitalization were attenuated and generally non-significant.

Most animal contact or household variables did not show strong trends for association with high-level resistance patterns, except that greater household size was negatively associated with ciprofloxacin resistance (PR 0.79 for household sizes over two, *p* = 0.04). However, 29 (76%) of the older case-patients lived in households with, at most, one other person. No significant trends in multidrug resistance over time (August 2009 to March 2010) were found (results not shown).

We also performed a sensitivity analysis examining inclusion of indirect effects from household members. We created a dummy variable in which we assigned: (2) the patient reported hospitalization and/or antimicrobial use within the past six months, (1) the patient reported that a he or she had not been hospitalized or taken antimicrobial drugs, but a household member had done so in the last six months, or (0) neither patient nor household member reported a history of hospitalization or antimicrobial use in the past six months. Patient history of hospitalization or antimicrobial use was associated with a 1.51 [95% CI: 1.01–2.25] fold higher risk of multidrug resistance (MDR4) in the patient’s isolate, and this was statistically significant (*p* = 0.05). Household member risk in the absence of patient risk was not associated with multidrug resistance (PR 0.46 [95% CI: 0.08–2.64], *p* = 0.38), but only four patients reported household member risk in the absence of patient risk.

### Associations in CA-MRSA Case-patients

**Figure 2:**
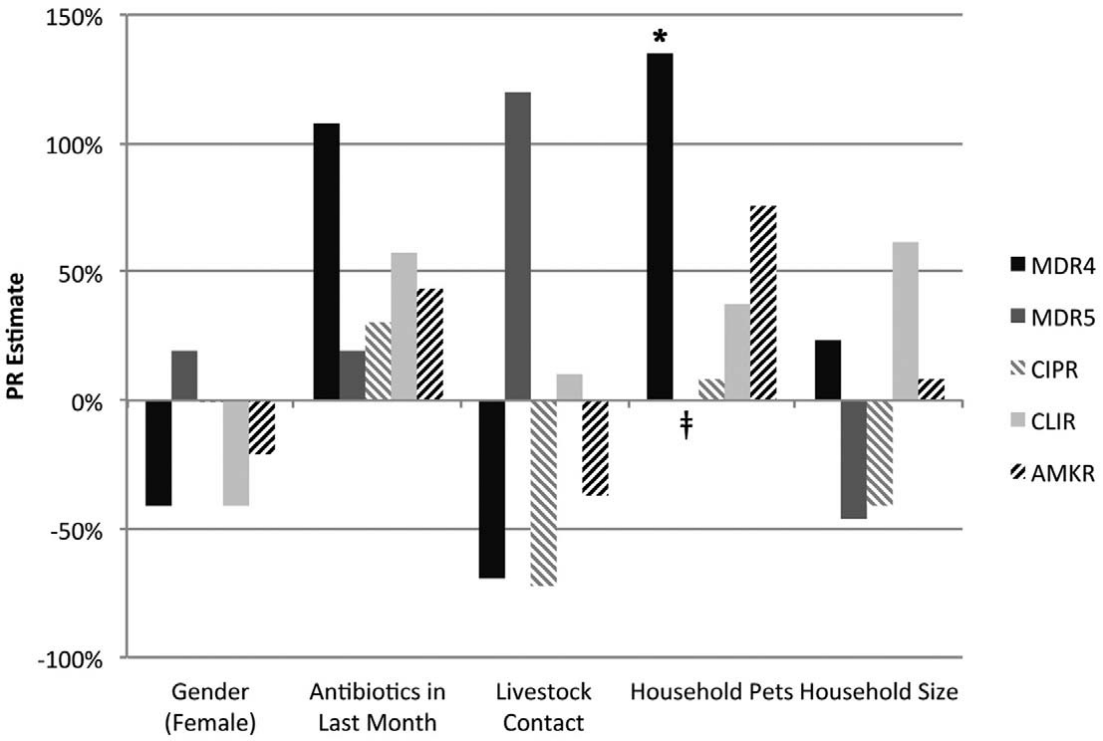
Risks for antimicrobial resistance in CA-MRSA case-patients of working age (18–65), *n = 27.* *p<0.10. No estimates were statistically significant at the *p*<0.05 level. ‡ A PR could not be estimated for MDR5 (high multi-drug resistance, 5+ classes of antimicrobial) for household pet presence due to a 0 stratum. Antimicrobial resistance patterns: MDR4: nonsusceptibility to four or more classes of antimicrobial drug; MDR5 (“high multidrug resistance”): high-level (complete) resistance to five or more classes of antimicrobial drug; CIPR: high-level resistance to ciprofloxacin; CLIR: high-level (complete) resistance to clindamycin, including inducible resistance; AMKR: high-level (complete) resistance to amikacin.

Because of interest in evaluating risks for colonization with multidrug-resistant MRSA among community associated (CA-)MRSA case-patients, we also performed analysis restricted to PSHMC case-patients who had reported no history of hospitalization in the past six months. We stratified this analysis by age due to *a priori* concerns with potential differences in risk factors for community acquisition of MRSA by age, and also based on evidence from the data that age independently was associated with increases in risk for antimicrobial resistance. We evaluated 27 CA-MRSA case-patients of working age (18–65) and 18 CA-MRSA case-patients over the age of 65. Due to statistical properties (strata with zero observations) in the older sub-cohort, few associations could be estimated for case-patients over the age of 65, but estimates that could be made sometimes were associated in the opposite direction from those of working-age CA-MRSA case-patients, supporting our decision to stratify by age. For working-age CA-MRSA case-patients, **Figure 2** presents the predicted PR estimates according to gender, use of antimicrobials in the last month, contact with livestock, household pet contact, and human household size. Household pet contact was associated with a 2.35-fold higher prevalence of multidrug resistance (MDR4), but this association was not significant (*p* = 0.09). Although a PR could not be estimated for associations between MDR5 and household pet contact due to a zero stratum, data from other strata suggested that this was a high-risk category. Estimates of risk with livestock were heavily influenced by a single case patient reporting direct contact with livestock who had an isolate resistant to beta-lactam, erythromycin, ciprofloxacin, clindamycin, and amikacin antimicrobials.

### Length of Stay Outcome

Geometric mean length of stay (LOS) was 5.73 days, ranging from 1.25 to over 45 days. On average, among PSHMC case-patients, having a multidrug-resistant isolate (MDR4) on admission was associated with a 1.5-day [95% CI: 1.11–1.97] increase in subsequent length of stay (LOS) in unadjusted models, and this association was statistically significant (*p* = 0.008). LOS estimates were similar for having a clindamycin-resistant or ciprofloxacin-resistant isolate. When history of hospitalization within the prior six months was included in LOS models, the estimate attenuated to a 1.3-day [95% CI: 1.01–1.80] increase (*p* = 0.05) and for having a clindamycin resistant isolate attenuated to a 1.4-day [95% CI: 1.06–1.80] increase (*p* = 0.02); the association between ciprofloxacin resistance and LOS lost significance.

### NHANES Analysis

In the 2003–04 NHANES population, prevalence of multidrug resistance (MDR4) was 73%, but prevalence of high multidrug resistance (MDR5) was only 4%, precluding further analysis. Prevalence of clindamycin resistance (inducible or high-level resistance) was 63%. Susceptibility to amikacin and ciprofloxacin were not tested; prevalence of levofloxacin resistance was 46%. Age distribution was: *n* = 47 for under 18, *n* = 49 for 18–65, and *n* = 37 for over 65 years of age. **Table 3** provides results of unadjusted and adjusted models from 133 NHANES MRSA-colonized individuals (2003–04) and 88 PSHMC MRSA-colonized inpatients (2009–10). In both the NHANES 2003–04 and PSHMC 2009–10 populations, older age and history of hospitalization were positively associated with multidrug resistance. In the NHANES 2003–04 population, greater household size was negatively associated with multidrug resistance in MRSA isolates.

**Table 3:** Antimicrobial resistance by risk factor among MRSA isolates comparing data from NHANES 2003–04 and data from Penn State Hershey Medical Center 2009–10.

	NHANES 2003–04						PSHMC 2009–10					
	*n* = 133						*n* = 88					
	Multidrug Resistance (MDR4)			Clindamycin Resistant(CLIR)			Multidrug Resistance (MDR4)			Clindamycin Resistant (CLIR)		
	*4+ classes of* *non-susceptibility*			*high-level resistance*			*4+ classes of* *non-susceptibility*			*high-level resistance*		
Prevalence	73%			63%			73%			58%		
	PR	95% CI	p-value	PR	95% CI	p-value	PR	95% CI	p-value	PR	95% CI	p-value
**Gender (male is ref)**												
Unadjusted	1.00	0.81–1.23	0.98	1.04	0.80–1.35	0.78	0.97	0.74–1.27	0.83	0.98	0.68–1.41	0.90
Adjusted	0.98	0.81–1.20	0.87	1.01	0.79–1.30	0.91	0.93	0.71–1.22	0.60	0.91	0.64–1.32	0.63
**Age (18–65 is ref)**												
Over 65, Unadjusted	**1.61**	**1.24–2.09**	**<0.001**	**1.82**	**1.34–2.48**	**<0.001**	1.16	0.90–1.49	0.25	*1.37*	*0.96–1.95*	*0.08*
Over 65, Adjusted	**1.36**	**1.03–1.78**	**0.03**	**1.46**	**1.03–2.04**	**0.03**	1.20	0.93–1.55	0.17	*1.40*	*0.97–2.02*	*0.07*
Under 18, Unadjusted	*1.30*	*0.97–1.75*	*0.08*	1.17	0.80–1.71	0.41	–			*–*		
Under 18, Adjusted	**1.44**	**1.05–1.97**	**0.02**	1.31	0.89–1.93	0.17	–			*–*		
**Hospitalization (>1 year is ref)**												
Within 1 year, unadjusted	**1.30**	**1.08–1.57**	**0.005**	**1.53**	**1.22–1.92**	**<0.001**	1.22	0.90–1.67	0.20	1.13	0.76–1.68	0.54
Within 1 month, adjusted	**1.22**	**1.00–1.48**	**0.05**	**1.36**	**1.07–2.04**	**0.01**	1.23	0.91–1.66	0.19	1.13	0.77–1.67	0.53
Household Size* (under 2 is ref)												
Unadjusted	**0.79**	**0.65–0.96**	**0.02**	**0.68**	**0.53–0.88**	**0.003**	1.05	0.81–1.36	0.71	0.95	0.65–1.39	0.81
Adjusted	0.93	0.73–1.20	0.59	*0.76*	*0.55–1.06*	*0.10*	1.10	0.85–1.41	0.47	1.04	0.70–1.52	0.86

Adjusted models include gender, age, hospitalization, and household size.

Conversely, among the 9004 NHANES participants tested in 2003–04 for any nasal colonization with *S. aureus* (MRSA and MSSA combined), odds were 1.32 fold higher [95% CI: 1.10–1.57] for colonization among participants who lived in larger households (*p* = 0.005). This association remained significant in survey-weighted, adjusted models controlling for gender, age, and hospitalization within the prior year (OR 1.37 [95% CI: 1.15–1.62], *p* = 0.001).

## Discussion

In this cohort of MRSA case-patients from primarily rural and suburban Pennsylvania, most isolates were susceptible to quinupristin-dalfopristin (Synercid); chloramphenicol; tetracycline; gentamicin; and trimethoprim/sufamethoxazole. Low rates of resistance to tetracycline and trimethoprim/sulfamethoxazole have been found in clinical MRSA isolates in other studies [34], [35]. Overall, most isolates were resistant to erythromycin and ciprofloxacin. Other studies have shown high levels of erythromycin resistance in MRSA isolates [9], [35]–[37] and increasing resistance to fluoroquinolones [9], [37], including in NHANES [32]. However, the 82% rate of resistance to ciprofloxacin in this cohort was much higher than rates of 40–45% reported in a North American hospital-based prevalence study in 2001–02 [35] or 50–55% rates reported through NHANES for 2001–2004 [32]. The 58% overall rate of resistance to clindamycin in this study was higher than the 30–35% reported in the 2001–02 North American hospital data [35], but was comparable with national trends of inducible or constitutive resistance in isolates collected between 2001–2004 [32].

In this study, recent hospitalization was associated with patient risk for carrying a multidrug-resistant strain of MRSA, and carriage of such strains was associated with increases in length of stay among case-patients admitted to the hospital for medical reasons unrelated to colonization status. Patients with prior hospital contact were more likely to be colonized with multidrug resistant isolates, and patients with prior hospital contact might have been a biased group with more severe medical conditions. However, inclusion of history of hospitalization in models did not negate the association.

When patients with recent hospital contact were excluded from PSHMC analysis and models were limited to working-age case-patients, potential but non-significant associations with animal contact emerged, particularly with dogs and cats. We did not collect information on antimicrobial use in pets, which might be a source of selective pressure for antimicrobial resistance in households. A prior study by Lin and colleagues described five MRSA isolates in animals from PA, with four of canine origin, from a study of clinical isolates collected during 2006–08 [38]. These isolates were of human HA-MRSA MLST types (primarily CC5) and displayed high levels of antimicrobial non-susceptibility to erythromycin, and additionally to veterinary fluoroquinolones, *e.g.,* enrofloxacin, (clindamycin was not tested) [38]. Clinical veterinary data analyzed by Rankin, Morris and colleagues from the Matthew J. Ryan veterinary hospital in Philadelphia demonstrated 100% resistance to clindamycin, ciprofloxacin, and erythromycin among nine PVL-positive clinical MRSA isolates from tested dogs and cats [39], and high clindamycin (72%), erythromycin (85%) and fluoroquinolone (90%) resistance among all 39 MRSA companion animal isolates submitted for 2003–04 [40]. The epidemic of MRSA in humans has been speculated to drive the parallel epidemic in companion animals, in part because companion animals tend to carry strains of MRSA typically associated with human transmission, and in part because households may be points of transmission between humans and companion animals [41]–[44]. The pilot data we report here may suggest that companion animals can serve as sources of antimicrobial resistance (potentially by harboring drug-resistant *S. aureus*, veterinary pathogens methicillin-resistant *S. pseudintermedius* and *S. schleiferi*, and other staphylococci [15]), but conclusions are limited by the small sample size and cross-sectional nature of the study. Longitudinal studies are needed to evaluate the potential association between animal contact and antimicrobial resistance in MRSA strains found in humans.

This study evaluated isolates from nasal colonization of newly-admitted hospital inpatients, a group likely to have greater prior exposure to healthcare settings and antimicrobial use than an outpatient population. Due to this potential bias, we also evaluated data from NHANES 2003-04 for similar risk factors. Typically, associations with antimicrobial resistance were in the same direction, although somewhat stronger in the NHANES data, for older age, hospitalization history, and smaller household size risk factors among individuals with MRSA colonization. However, larger household size was a risk factor for *S. aureus* nasal colonization in the U.S. population generally, indicating that household size may play a complicated role in *S. aureus* and MRSA epidemiology. Evaluation of MRSA-colonized individuals demonstrated higher risk for isolate antimicrobial resistance in smaller households, although this association was attenuated when models accounted for gender, age, and history of hospitalization. Comparisons between PSHMC and NHANES data are limited by differences in methodology and potential temporal trends in MRSA epidemiology between 2003–04 (NHANES) and 2009–10 (PSHMC). Future NHANES surveys of national *S. aureus* colonization should consider including survey questions on pet ownership and animal contact.

In conclusion, carriage of ciprofloxacin-resistant, clindamycin-resistant, MDR4 and MDR5 MRSA was associated with prior history of hospitalization, but not with history of antimicrobial use, in MRSA case-patients colonized at admission to a tertiary care center. Similarly, prior history of hospitalization, older age (over 65), younger age (under 18), and small household size were risk factors for multidrug resistance and resistance to clindamycin in MRSA-colonized individuals who participated in NHANES 2003–04. Colonization with multidrug-resistant isolates was associated with increases in subsequent length-of-stay for patients in the hospital, with economic and clinical implications. Animal contact, particularly with household pets, may be an emerging risk factor for isolate antimicrobial resistance in case-patients lacking recent history of hospitalization, but this potential association should be confirmed with larger studies.
